# ApoM and Major Adverse Cardiovascular Events in Chronic Kidney Disease: A Prospective Cohort Study

**DOI:** 10.1161/ATVBAHA.124.322367

**Published:** 2025-03-06

**Authors:** Julia T. Stadler, Andrea Borenich, Line Stattau Bisgaard, Sasha S. Bjergfelt, Sarunja Vijayakumar, Line Melholt, Insa E. Emrich, Ditte Hansen, Susanne Bro, Christina Christoffersen, Gunnar H. Heine, Gunther Marsche

**Affiliations:** 1 Division of Pharmacology, Otto Loewi Research Center for Vascular Biology, Immunology and Inflammation (J.T.S., G.M.), Medical University of Graz, Austria.; 2 Institute for Medical Informatics, Statistics and Documentation (A.B.), Medical University of Graz, Austria.; 3 Institute of Pharmaceutical Sciences, Department of Pharmacognosy, University of Graz, Austria (J.T.S.).; 4 Departments of Clinical Biochemistry (L.S.B., S.V., L.M., C.C.), Copenhagen University Hospital, Rigshospitalet, Denmark.; 5 Nephrology (S.S.B., S.B.), Copenhagen University Hospital, Rigshospitalet, Denmark.; 6 Departments of Biomedical Sciences (L.S.B., S.S.B., C.C.), University of Copenhagen, Denmark.; 7 Clinical Medicine (D.H.), University of Copenhagen, Denmark.; 8 Faculty of Medicine, Saarland University, Homburg/Saarbrücken, Germany (I.E.E., G.H.H.).; 9 Department of Nephrology, Copenhagen University Hospital, Herlev-Gentofte, Denmark (D.H.).; 10 Department of Nephrology, Agaplesion Markus Krankenhaus, Frankfurt am Main, Germany (G.H.H.).

**Keywords:** apolipoproteins M, cholesterol, humans, renal insufficiency, chronic, risk factors

## Abstract

**BACKGROUND:**

Cardiovascular disease is the leading cause of mortality in patients with chronic kidney disease (CKD). APOM plays a critical role in reverse cholesterol transport by facilitating the formation of pre-β-HDL (high-density lipoprotein) and enabling the binding of S1P (sphingosine-1-phosphate) to HDL, a complex involved in several antiatherogenic processes. In this study, we sought to investigate the potential association between plasma APOM levels and the risk of adverse cardiovascular outcomes in individuals with CKD.

**METHODS:**

Plasma APOM levels were quantified using a sandwich ELISA-based assay. Plasma S1P levels were measured by high-performance liquid chromatography. The primary end point was a composite of major adverse cardiovascular events (MACE) and all-cause mortality.

**RESULTS:**

In this secondary analysis of the CARE FOR HOMe study (Cardiovascular and Renal Outcome in CKD 2–4 Patients–The Fourth Homburg Evaluation), 463 nondialysis patients with CKD stages G2 to G4 were included. Plasma APOM levels exhibited a significant inverse association with the risk of MACE (standardized hazard ratio, 0.60 [95% CI, 0.49–0.75]; *P*<0.001) and all-cause mortality (standardized hazard ratio, 0.63 [95% CI, 0.48–0.83]; *P*<0.001). This inverse association with MACE remained robust after adjusting for established cardiovascular and renal risk factors. These findings were further corroborated in an independent cohort of 822 patients with CKD from the Copenhagen CKD study. Plasma S1P levels showed an inverse association with MACE in univariable analyses; however, this relationship lost statistical significance after multivariable adjustments.

**CONCLUSIONS:**

Our findings demonstrate a significant association between low plasma APOM levels and an increased risk of MACE in patients with CKD. These results suggest that APOM may play a role in cardiovascular protection in this vulnerable population.

**Highlights**

- Plasma APOM is reduced in advanced chronic kidney disease, independent of HDL (high-density lipoprotein) cholesterol levels.
- Reduced plasma APOM levels are independently associated with major adverse atherosclerotic events in patients with chronic kidney disease.
- Findings were corroborated in an independent cohort of patients with chronic kidney disease from the Copenhagen chronic kidney disease study.

Chronic kidney disease (CKD) impacts >10% of the global population, and this prevalence is steadily increasing.^1^ Factors contributing to this rising incidence include the higher prevalence of type 2 diabetes, hypertension, and the aging population, as well as challenges such as limited diagnostic options and therapeutic inertia in the earlier stages of CKD.^2–4^ It is now recognized that a high rate of fatal and nonfatal cardiovascular events already occurs in patients with earlier stages of CKD. Renal disease significantly disrupts lipoprotein metabolism, leading to various lipid abnormalities.^5^ In advanced CKD, which is associated with inflammation and oxidative stress, HDL (high-density lipoprotein) is often dysfunctional and less protective, sometimes even exhibiting proatherogenic characteristics.^6–10^ One of the CKD-related compositional changes of HDL is a reduction of APOM.^11^ This apolipoprotein belongs to the lipocalin family and is primarily secreted by the liver and kidneys.^12^ While it is mainly associated with HDL particles, it is also found in small quantities in triglyceride-rich lipoproteins.^13^ A considerable body of research has demonstrated the multifaceted beneficial properties of APOM, including its anti-inflammatory and antiatherogenic activities.^13,14^ APOM is required for pre-β-HDL formation and cholesterol efflux to HDL and protects against atherosclerosis.^15,16^ The structure of APOM contains a hydrophobic binding pocket that binds the bioactive lipid S1P (sphingosine-1-phosphate). The APOM/S1P complex has been demonstrated to reduce inflammation by inhibiting monocyte adhesion to the endothelium and maintaining endothelial barrier integrity.^17^ A meta-analysis identified a significant association between a polymorphism in the human *APOM* gene and coronary heart disease risk within the Chinese population.^18^ Moreover, a recent study demonstrated an independent association between lower circulating APOM levels and increased mortality in a non-CKD population of patients with chronic heart failure,^19^ highlighting the potential clinical significance of APOM.

Based on the established anti-inflammatory and cardioprotective effects attributed to APOM, we sought to determine whether reduced plasma levels of APOM may be associated with increased susceptibility to major adverse cardiovascular events (MACE) in patients with CKD.

## Materials and Methods

The data that support the findings of this study are available from the corresponding author upon reasonable request.

### Study Populations

The primary analysis utilized data from participants in the CARE FOR HOMe study (Cardiovascular and Renal Outcome in CKD 2–4 Patients–The Fourth Homburg Evaluation). To confirm the findings, an independent cohort, the Copenhagen CKD cohort, was used as a validation source.

### CARE FOR HOMe Study

The CARE FOR HOMe study is a prospective cohort study that enrolled 526 patients with CKD Kidney Disease: Improving Global Outcomes stages G2 to G4, determined by their estimated glomerular filtration rate (eGFR) between 2008 and 2015.^20^ Patients in the G2 stage have an eGFR between 60 and 89 mL/min per 1.73 m². Those in the G3a stage have an eGFR of 45 to 59 mL/min per 1.73 m², while G3b corresponds to an eGFR of 30 to 44 mL/min per 1.73 m². Patients in the G4 stage exhibit severely decreased kidney function, with an eGFR ranging from 15 to 29 mL/min per 1.73 m². Our analysis included 463 patients selected based on the availability of sufficient material for plasma APOM measurements. The CARE FOR HOMe study aimed to investigate the risk factors for CKD progression and identify patients at high risk for cardiovascular complications. All patients were treated at the renal outpatient clinic of the Saarland University Medical Centre in Homburg, Germany. Exclusion criteria included acute kidney injury, transplant recipients, pregnant women, participants under 18 years of age, patients receiving systemic immunosuppressive medications, and those with apparent infections. This study was conducted in accordance with the Declaration of Helsinki and was approved by the local Ethics Committee of the Medical Association of Saarland (Faktoreistraße 4, 66111 Saarbrücken, Germany; ethics committee code: 08/10). All patients provided written informed consent before inclusion.

At baseline, fasted blood samples were collected from participants, and a standardized questionnaire was utilized to collect data about the medical history. Confirmation of prevalent cardiovascular disease (CVD) was conducted through chart review, encompassing a history of coronary artery angioplasty/stenting/bypass surgery, myocardial infarction, carotid endarterectomy/stenting, nontraumatic lower extremity amputation, lower limb artery angioplasty/stenting/bypass surgery, and major stroke.

Patients were annually invited for a follow-up examination at the study center. The primary end point MACE was predefined as the first occurrence of any of the listed cardiovascular events: myocardial infarction, coronary artery angioplasty/stenting/bypass surgery, major stroke, carotid endarterectomy/stenting, nontraumatic lower extremity amputation and lower limb artery angioplasty/stenting bypass surgery, and all-cause mortality. As a secondary end point, all-cause mortality was analyzed separately.

### Copenhagen CKD Validation Cohort

The Copenhagen CKD cohort is a prospective, observational investigation focused on assessing cardiovascular risk in patients with CKD. Details of the study population have been published previously.^21^ For the present analysis, nondialysis patients with CKD stages G2 to G4 were selected, yielding a total of 822 participants. Patients were recruited from nephrology outpatient clinics at the Department of Nephrology, Copenhagen University Hospital, Rigshospitalet, and Herlev and Gentofte Hospital, between October 2015 and December 2018. The eGFR and corresponding baseline CKD stage were determined using the CKD Epidemiology Collaboration equation.^22^ Medical and demographic information at baseline were obtained from electronic medical records and patient interviews, whereas anthropometric and blood pressure measurements were collected during a physical examination. The prevalence of hypertension and hypercholesterolemia has been defined previously.^21^ The study followed the Declaration of Helsinki II principles and was approved by the Danish Scientific Ethical Committee (H-3-2011-069) and the Danish Data Protection Agency (30-0840). All participants signed a written informed consent before inclusion. The primary end point MACE was defined as a composite of cardiovascular events and all-cause mortality. Cardiovascular events were defined as myocardial infarction, percutaneous coronary intervention, coronary bypass surgery bypass surgery, ischemic stroke, carotid endarterectomy or stenting, nontraumatic amputation, carotid endarterectomy or stenting, nontraumatic lower limb amputation, lower limb bypass grafting, arterial bypass grafting, and percutaneous transluminal angioplasty of a lower limb. All-cause mortality was defined as death from any cause, occurring in or out of hospital. The outcome data were analyzed by reviewing the electronic medical records.

### Plasma APOM Quantification

Plasma APOM levels were quantified using a previously described sandwich ELISA-based assay.^23^ Briefly, a high-binding Costar 96-well plate (Corning, New York, NY) was coated overnight with capture antibody against human APOM (M03, Research Resource Identifiers [RRID]: AB_1112555, 50 μL per well; Abnova, Taipei, Taiwan) in a concentration of 5 μg/mL diluted in Tris-buffered saline (TBS). The membrane was blocked with 2% BSA in TBS for 2 hours. To unfold APOM, 10 mL of plasma was incubated with 90 mL of 55 mmol/L dithiothreitol in 0.2 mol/L sodium phosphate buffer for 10 minutes at 30 °C and 1100 revolutions/min, protected from light. Subsequently, 100 mL of 0.6 mol/L iodoacetamide in 0.02 mol/L sodium phosphate buffer was added and incubated at room temperature for 1 hour at 600 revolutions/min. Diluted samples (1:50 in TBS with 1% BSA) were then transferred to a precoated ELISA plate and incubated overnight at room temperature. The plate was washed 4× with 0.1% Triton X-100 in TBS and incubated with a 1:1000 dilution of human APOM detection antibody (EPR2904, RRID: AB_2049169; Abcam, Cambridge, United Kingdom) in TBS with 1% BSA and 2% Triton X-100 for at least 3 hours.

After washing, the plate was incubated with a 1:2000 dilution of horseradish peroxidase–conjugated anti-rabbit IgG in TBS with 1% BSA and 0.1% Triton X-100 for at least 2 hours. Following another wash, the plate was incubated with a development solution for 8 to 10 minutes and the reaction was stopped with 1 mol/L sulfuric acid. Absorbance was measured at 492 nm using an ELISA reader. The APOM concentration in the samples was determined from a standard curve included in each measurement. The assay’s intra-assay coefficient of variation was 8.5% and the inter-assay coefficient of variation was 4.3%.

### Plasma S1P Measurements

S1P content in the plasma was measured by high-performance liquid chromatography (HPLC) as described elsewhere.^14^ In brief, the S1P extraction was performed with chloroform-methanol in a 2-step procedure followed by derivatization with 2,3-naphthalene-carboxaldehyde. Subsequently, derivatized samples were analyzed with Agilent 1290 HPLC (Agilent, Santa Clara, CA) using a Synergi4u Fusion-RP 80A column (30×2.0 mm; Phenomenex) with a flow of 0.5 mL/min. The used solvent phases were as follows: mobile phase composed of 85% acetonitrile with 15% methanol (HPLC grade; Rathburn Chemicals, Ltd, Walkerburn, United Kingdom) and aqueous phase composed of 20 mmol/L H_2_KPO_4_ (pH 4.8) and 15% methanol (HPLC grade; Merk, Darmstadt, Germany). The separation was achieved by using a gradient of the mobile phase: 0 to 6 minutes, 47.5%; 6 to 9 minutes, 47.5%–87.5%; 9 to 10 minutes, 87.5%; 10 to 12 minutes, 87.5%–47.5%; and 12 to 15 minutes, 47.5%. Using the same control in each extraction and measurement, an inter-assay coefficient of variation of 13.9 was calculated.

### Statistical Analyses

Patients were stratified into tertiles based on their plasma APOM levels, and various clinical parameters were compared across these groups. The clinical characteristics were summarized using mean (SD) for normally distributed variables and median (Q1, Q3) for variables with skewed distributions. Categorical variables are presented as number of patients (percentages). The distribution of variables was assessed by examining QQ plots and the Shapiro-Wilk test. Differences between groups were calculated using the Kruskal-Wallis test and Pearson χ^2^ test. Kaplan-Meier plots for end points of MACE and all-cause mortality were computed, followed by log-rank tests to assess the association of plasma APOM with adverse outcomes in CKD. Cox regression models were used with standardized APOM levels, and hazard ratios were expressed per increase of 1 SD for the end points. Restricted cubic spline analyses were performed to visualize the univariable and adjusted association of continuous APOM levels with the risk of adverse outcomes. Correlations between plasma APOM and clinical parameters were calculated using Spearman correlation coefficients. A *P* value of <0.05 was considered significant. Statistical analyses were performed using GraphPad Prism 9.0.0, SPSS Statistics 26, and R, version 4.3.3.

## Results

### Baseline Characteristics of the CARE FOR HOMe Study Participants

Patients of the CARE FOR HOMe study were stratified into tertiles based on their plasma APOM levels (Table). Most patients were in CKD stage 3a (33%) and CKD stage 3b (28%). The mean age of the participants at the study entry was 65 years, with an SD of 12 years. Overall, 33% of the cohort had prevalent CVD and 39% had been diagnosed with diabetes.

**Table.**
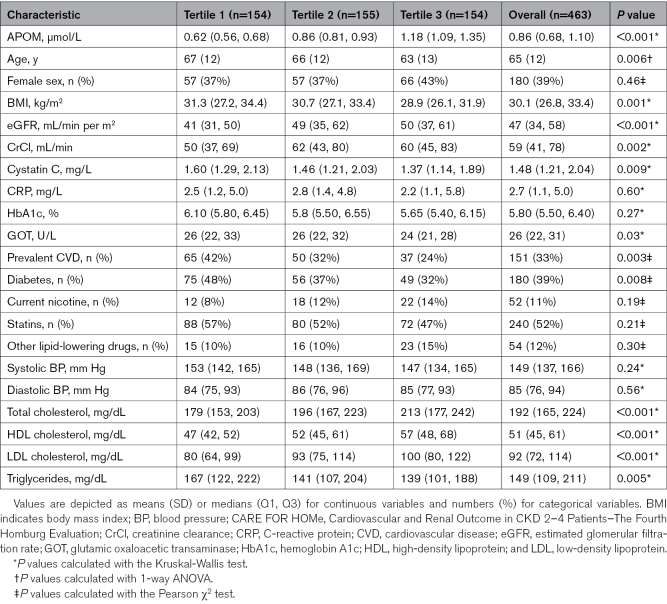
Baseline Characteristics of the CARE FOR HOMe Study Cohort, Stratified by APOM Tertiles

Notably, patients in the lowest tertile of APOM levels were older, had a higher body mass index, and exhibited lower baseline renal function. Additionally, these individuals demonstrated a higher prevalence of cardiovascular comorbidities and a greater incidence of diabetes. Plasma lipid levels varied significantly across APOM tertiles, with the lowest tertile showing reduced levels of total cholesterol, LDL (low-density lipoprotein) cholesterol, HDL cholesterol (HDL-C), and elevated triglyceride levels. No significant differences were observed in CRP (C-reactive protein) levels, hemoglobin A1c levels, or blood pressure measurements across the tertiles.

### Plasma APOM Levels Are Reduced in Patients With Advanced CKD

**Figure 1.**
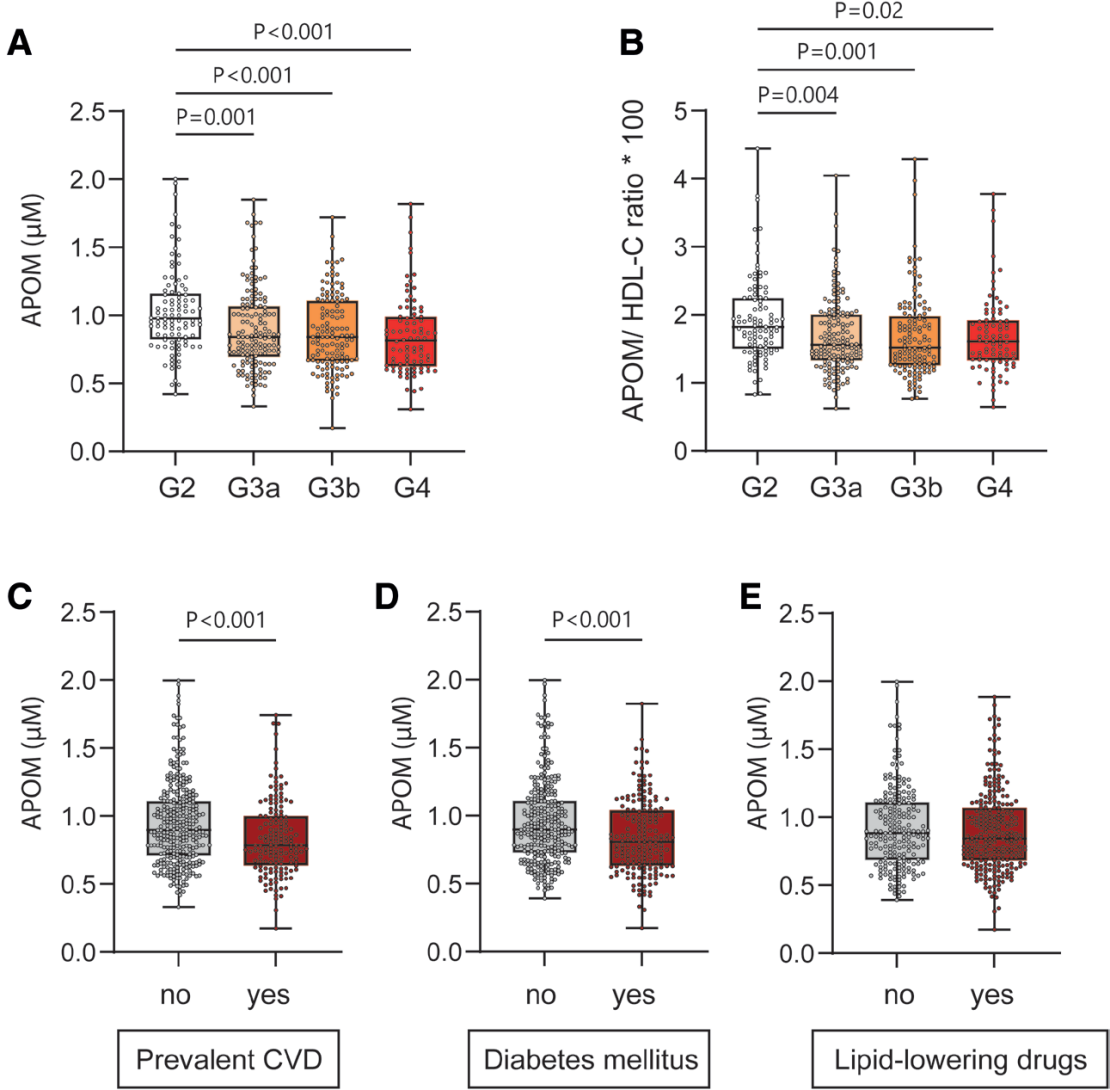
**Plasma levels of APOM are reduced in patients with advanced estimated glomerular filtration rate categories in the CARE FOR HOMe study (Cardiovascular and Renal Outcome in CKD 2–4 Patients–The Fourth Homburg Evaluation).** Plasma levels of APOM in patients stratified by severity stages of chronic kidney disease (**A**). APOM/high-density lipoprotein cholesterol (HDL-C) ratio to correct for differences in HDL-C levels (**B**). Association of plasma APOM with prevalent cardiovascular disease (CVD; **C**), prevalent diabetes (**D**), and lipid-lowering medication (**E**). Data are presented as boxplots, displaying the median, interquartile range, and minimum and maximum values. Differences between groups were analyzed by the Kruskal-Wallis test followed by post hoc Dunn multiple comparison test.

To investigate the association between APOM levels and kidney function, we stratified patients of the CARE FOR HOME study by CKD severity stage, according to Kidney Disease: Improving Global Outcomes.^20^ Notably, plasma APOM levels were significantly reduced in patients with more advanced CKD stages, as shown in Figure 1A.

Given that APOM is predominantly bound to HDL in the plasma, we explored whether the reduction in APOM levels was related to a potential decrease in HDL. To assess this, we calculated the ratio of APOM to HDL-C (multiplied by 100). This analysis revealed that APOM levels remained diminished with increasing CKD severity, indicating that the reduction in APOM is not solely attributable to changes in HDL-C (Figure 1B).

Further analysis of the association between prevalent comorbidities and medication use with plasma APOM levels showed decreased APOM levels among patients with prevalent CVD and diabetes (Figure 1C and 1D). However, no significant difference in APOM levels was observed among patients receiving lipid-lowering medications (Figure 1E).

**Figure 2.**
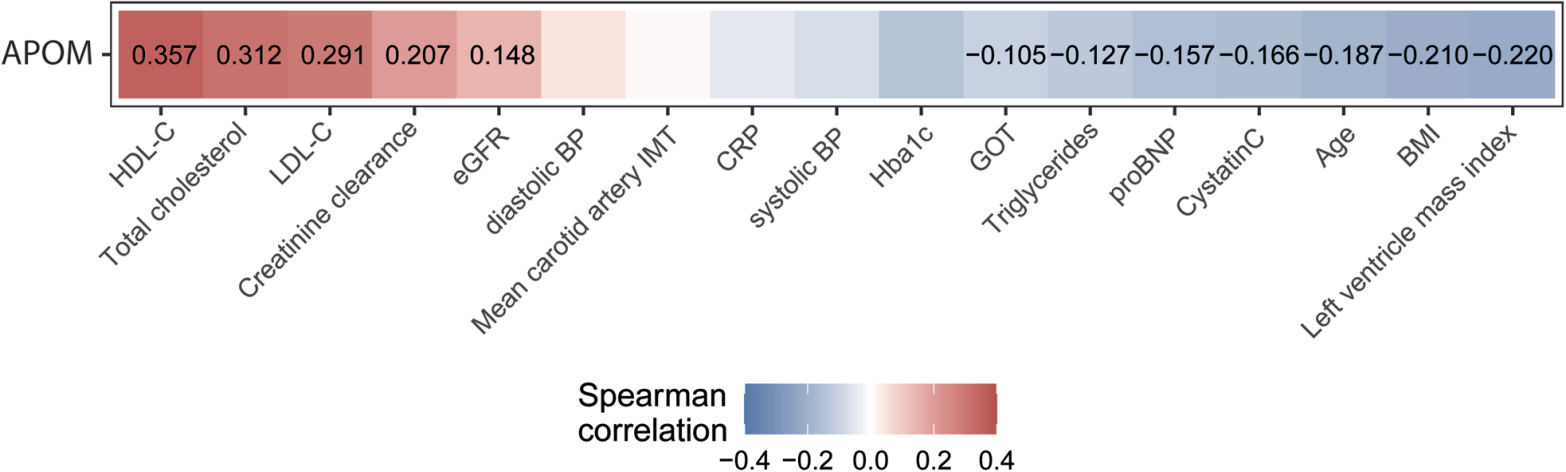
**Correlation of plasma APOM levels with clinical characteristics in patients with chronic kidney disease (CKD) of the CARE FOR HOMe study (Cardiovascular and Renal Outcome in CKD 2–4 Patients–The Fourth Homburg Evaluation).** Each cell of the heatmap represents a pairwise Spearman correlation between the 2 parameters indicated in the respective row and column. Significant correlations (*P*<0.05) are indicated with the corresponding Spearman correlation coefficient. Nonsignificant correlations can still be inferred from the color but are not explicitly indicated. BMI indicates body mass index; BP, blood pressure; CRP, c-reactive protein; eGFR, estimated glomerular filtration rate; GOT, glutamic oxaloacetic transaminase; HbA1c, glycated hemoglobin; HDL-C, high-density lipoprotein cholesterol; IMT, intima-media thickness; LDL-C, low-density lipoprotein cholesterol; and proBNP, pro-B-type natriuretic peptide.

Next, we investigated whether the clinical characteristics correlated with plasma APOM levels (Figure 2). We conducted exploratory Spearman correlation analyses, which revealed that APOM levels were negatively correlated with age, body mass index, NT-proBNP (N-terminal pro-B-type natriuretic peptide), and left ventricular mass index. Additionally, plasma APOM levels were associated with markers of renal function, including eGFR, creatinine clearance, and cystatin C. APOM showed positive correlations with total cholesterol and LDL cholesterol. Notably, the strongest correlation for APOM was with HDL-C (r_S_=0.357; *P*<0.001).

### Low Plasma APOM Levels Are Associated With Adverse Outcomes in CKD

**Figure 3.**
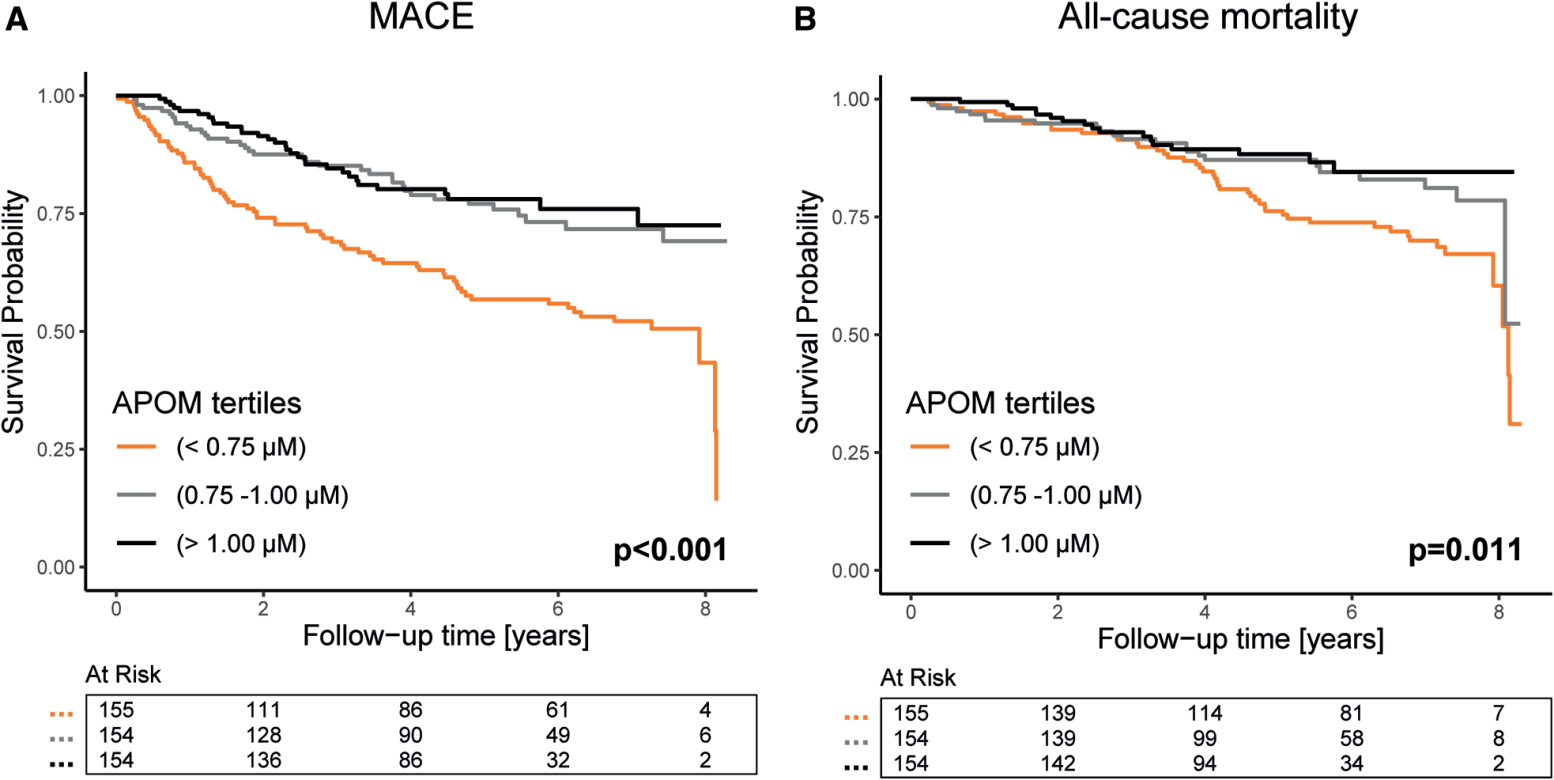
**Risk of adverse outcomes among patients with chronic kidney disease (CKD) of the CARE FOR HOMe study (Cardiovascular and Renal Outcome in CKD 2–4 Patients–The Fourth Homburg Evaluation) and tertiles of APOM plasma levels.** Kaplan-Meier plots with subsequent log-rank tests for risk of major atherosclerotic cardiovascular events (MACE*; **A**) and all-cause mortality (**B**) are shown. The number of patients at each time point is presented below the graph. *Composite of MACE, including death of any cause.

**Figure 4.**
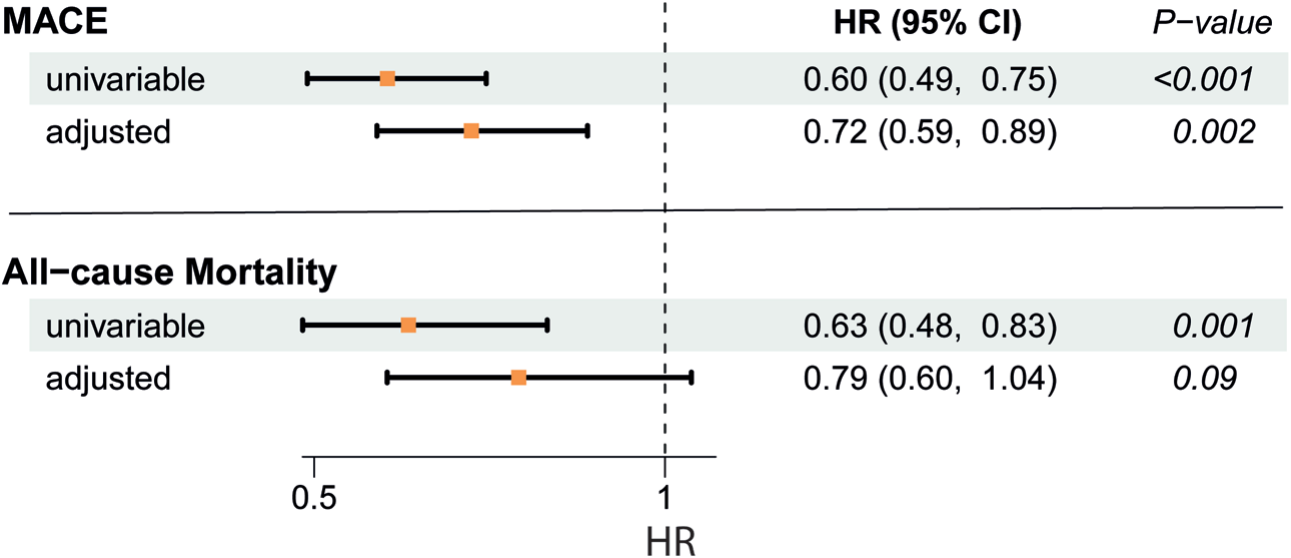
**Hazard ratios (HRs) per 1 SD increase and 95% CIs from Cox regression analyses.** Plasma APOM levels were standardized and used as continuous variables, and risk with major atherosclerotic cardiovascular events (MACE)* and all-cause mortality was assessed. The adjusted model includes age, sex, and estimated glomerular filtration rate. *Composite of MACE, including all-cause mortality.

During a mean follow-up period of 5.0±2.2 years, 139 (30%) patients of the CARE FOR HOMe study reached the primary end point of MACE, and 86 (19%) patients died from any cause. To assess the relationship between baseline plasma APOM levels and adverse outcomes, patients were stratified into tertiles based on their APOM levels. Kaplan-Meier analysis and log-rank tests revealed a significantly higher risk of both MACE and all-cause mortality in patients with the lowest APOM tertile (Figure 3). To evaluate the association of APOM with outcomes independent of other cardiovascular and renal risk factors, we performed multivariable Cox regression analyses (Figure 4). APOM levels were standardized for comparability and treated as a continuous variable. In univariable analysis, plasma APOM demonstrated a strong inverse association with the risk of MACE (hazard ratio per 1 SD increase, 0.60 [95% CI, 0.49–0.75]; *P*<0.001) and all-cause mortality (hazard ratio per 1 SD increase, 0.63 [95% CI, 0.48–0.83]; *P*<0.001). In an adjusted model accounting for age, sex, and eGFR, APOM remained significantly inversely associated with the risk of MACE (hazard ratio per 1 SD increase, 0.72 [95% CI, 0.59–0.89]; *P*=0.002). However, the significant association of APOM with all-cause mortality was attenuated (*P*=0.09). APOM remained an independent inverse predictor of MACE risk even after further adjustment for diabetes, hypercholesterolemia, smoking, and serum albumin (Table S2).

To examine the course of the relationship between plasma APOM levels and outcomes, cubic spline plots were generated from Cox regression analyses (Figure S2). Notably, lower plasma APOM levels were associated with increased risk for the outcomes, whereas higher APOM levels suggested a protective effect.

APOM acts as the physiological carrier of S1P on lipoproteins.^14^ To investigate whether the observed associations between APOM and study end points extend to S1P, we measured plasma S1P levels in a subset of 421 patients with available samples. Stratifying patients by CKD severity, we found no significant reduction in S1P levels with advancing CKD stages (Figure S1). Spearman correlation analyses of S1P with plasma APOM showed a significant, albeit weak, correlation (r_S_=0.16; *P*<0.001).

In univariable Cox regression analysis, S1P alone was associated with MACE (*P*=0.002) and tended to be associated with all-cause mortality (*P*=0.05). However, this significance was lost after adjusting for other risk factors (Table S1).

### Prognostic Implications of APOM Compared With HDL-C

**Figure 5.**
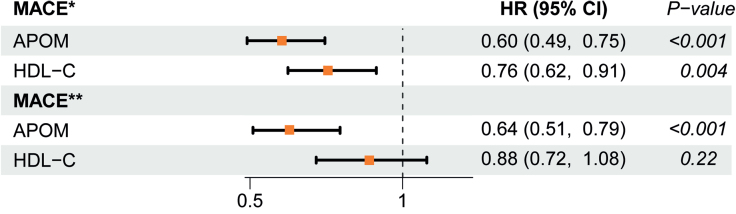
**APOM, but not high-density lipoprotein cholesterol (HDL-C), is inversely associated with the risk of major adverse cardiovascular events (MACE) in the CARE FOR HOMe study (Cardiovascular and Renal Outcome in CKD 2–4 Patients–The Fourth Homburg Evaluation).** Hazard ratios per 1 SD increase and 95% CIs. HR indicates hazard ratio. *Univariable analyses. **Analyses adjusted for either APOM or HDL-C.

Previous studies have demonstrated that the majority of plasma APOM is associated with HDL (90%–95%), with only a small proportion bound to triglyceride-rich lipoproteins.^11,13^ To determine whether the relationship between APOM and MACE is independent of its association with HDL-C, we performed Cox regression models evaluating the relationship between APOM and outcomes both with and without adjustments for HDL-C in the CARE FOR HOMe study (Figure 5). While APOM was inversely associated with the risk of both all-cause mortality and MACE, HDL-C was only inversely related to the risk of MACE and did not demonstrate a similar association with mortality. In models including both APOM and HDL-C, only APOM remained significantly associated with the risks of MACE and all-cause mortality, and this relationship was not weakened by the adjustment for HDL-C.

### Replication in the Copenhagen CKD Cohort

The clinical characteristics of the Copenhagen CKD cohort participants are shown in Table S2. During a mean follow-up period of 3.87±1.06 years, 151 (18.4%) participants reached the end point of MACE. Univariable Cox regression analyses revealed that a 1-SD increase in APOM levels was associated with a 24% reduction in the risk of MACE (Table S3). To increase the number of events and enhance the overall statistical power, we combined the 2 CKD cohorts, yielding a total of 1285 patients, among whom 290 experienced MACE. Cox regression analyses of the adjusted model demonstrated a significant inverse association between plasma APOM levels and MACE (Table S4).

## Discussion

Cardiovascular events pose a significant burden in CKD, ranking as the leading cause of mortality in this population.^24^ Given the intricate interplay between renal dysfunction and cardiovascular complications, there is an urgent need for novel therapeutic targets to improve patient outcomes.

Here, we demonstrate that reduced plasma levels of APOM are associated with an increased risk of MACE in patients with CKD, independent of traditional cardiovascular and renal risk factors in the CARE FOR HOMe study, a well-characterized cohort with many years of follow-up, and a large number of recorded events. We further validated these findings in the independent Copenhagen CKD cohort, providing strong evidence that reduced plasma APOM levels are independently associated with an increased risk of MACE in patients with CKD.

Stratifying patients by eGFR in our initial analysis of the CARE FOR HOMe study revealed a decrease in plasma APOM levels in advanced CKD compared with early CKD stage G2, consistent with previous research showing that APOM decreases from CKD stage G3.^11^ The normal plasma concentration of APOM in healthy individuals is ≈0.9 µmol/L, similar to patients with only mildly reduced eGFR within CKD stage G2 in our cohort.^25,26^ Our analysis revealed a consistent decrease in plasma APOM with worsening CKD stage. Despite the strong association between APOM and HDL levels, the reduction in APOM persisted even after adjusting for HDL-C levels, suggesting that factors beyond HDL quantity contribute to this decline.^13^ Notably, patients with CKD with preexisting CVD had lower baseline APOM levels, similar to those observed in patients with diabetes.^11^

Spearman correlation analyses of baseline clinical characteristics further unveiled a negative correlation between plasma APOM levels with age and body mass index, consistent with results from previous research.^27^ Our findings further support the association between APOM and renal function, as evidenced by the positive correlation observed with eGFR. Interestingly, despite the established inverse relationship between APOM and inflammation,^28,29^ we did not observe a significant correlation of APOM with CRP levels in our study. This warrants further investigation to understand the potential context-specific factors influencing the observed association between declining APOM levels and CKD progression. Experimental animal studies have demonstrated that moderate overexpression of APOM can slow the development of atherosclerosis in mouse models prone to atherosclerosis.^16,30^ Consistent with the proposed cardioprotective potential of the APOM/S1P complex,^31^ our analysis revealed an inverse correlation between plasma APOM levels and both left ventricular mass index and the cardiac biomarker NT-proBNP. This suggests a potential role for APOM in mitigating cardiac remodeling and subsequent dysfunction in patients with CKD.

It has to be noted that extreme overexpression of APOM in LDL receptor knockout mice accelerated atherosclerosis, likely due to the concomitant increase in lipid levels.^32^

Several human studies have already suggested a relationship between low levels of APOM and CVD.^18,33^ Specifically, a single-nucleotide polymorphism rs805296, located in the promoter region of the *APOM* gene, which leads to reduced plasma levels of APOM, has been identified as a potential risk factor for CVD in a meta-analysis of epidemiological studies.^33^ In addition, several studies have suggested that APOM may serve as a potential prognostic marker for mortality in various diseases, including heart failure, breast cancer, and sepsis.^19,34,35^ However, in the general population, low plasma levels of APOM are not associated with an increased risk for CVD.^36^

In our study of patients with CKD, we found a strong association between low plasma APOM levels and the risk of the primary end point MACE. The association of APOM with the outcome of all-cause mortality initially showed significance in univariable Cox regression analysis; however, this significance was lost after adjusting for other risk factors. Therefore, our findings suggest a more specific association of APOM with MACE rather than all-cause mortality in our study cohort. Importantly, we confirmed the inverse association between APOM levels and MACE in an independent cohort, the Copenhagen CKD cohort. This association observed in patients with CKD is a novel finding and aligns with the protective role of APOM demonstrated in animal studies focused on atherosclerosis. CKD, especially in advanced stages, is associated with systemic oxidative stress, endothelial dysfunction, and inflammation, all of which contribute to atherosclerosis. Thus, reduced levels of APOM, with its antioxidant, endothelium-protective, and antiatherogenic properties,^15,16,37^ may contribute to the increased susceptibility to atherosclerosis seen in patients with CKD.

Notably, the majority of APOM is associated with HDL, and only a small proportion is bound to triglyceride-rich lipoproteins. However, only about 5% of HDL particles carry APOM.^14^ This makes APOM-containing HDL a distinct and specialized subclass of HDL particles. When we compared the prognostic potential of APOM with HDL-C by accounting for each other in Cox regression, only APOM remained significantly associated with MACE, demonstrating an association independent of plasma HDL-C levels.

S1P is a bioactive sphingolipid that acts as a ligand for APOM.^14^ It plays a crucial role in vascular homeostasis by activating its G-protein–coupled receptors. Specifically, S1P has been demonstrated to enhance endothelial barrier function,^38,39^ induce vasodilator production,^40,41^ and inhibit endothelial inflammation.^17^ To investigate whether our observed associations of APOM with MACE in patients with CKD extend to S1P, we assessed S1P plasma levels in a subset of 421 patients by HPLC. S1P levels were not significantly reduced in patients with advanced CKD but were weakly correlated with plasma APOM levels. Previous studies have reported a strong correlation between APOM and S1P within isolated HDL particles.^19,42^ Consistent with our study’s findings, this correlation is much lower in the plasma^42^ given that S1P is not exclusively bound to HDL in circulation; it also binds to albumin, accounting for ≈30% of its binding.^14^ Survival analyses of plasma S1P levels and outcomes in the CARE FOR HOMe study revealed an inverse association with MACE in univariable analyses; however, this association was not significant after multivariable adjustments.

HDL containing APOM, which carries S1P and promotes endothelial function by activating S1P receptors, has been shown to enhance cholesterol efflux more effectively than HDL lacking APOM.^14,15^ This has led to the development of S1P carriers such as APOM-Fc because APOM alone has a relatively short half-life.^43^ Administration of APOM-Fc has demonstrated several beneficial effects, including reducing blood pressure in hypertensive mice, attenuating myocardial damage following ischemia/reperfusion injury, and reducing brain infarct volume in the middle cerebral artery occlusion model of stroke.^44^ Recently, a novel designer HDL, a fusion protein of APOM and apoA-I, has been developed that forms spherical nano-sized lipoprotein particles and provides additional benefits over previously engineered S1P carriers. This designer HDL chaperone binds multiple bioactive lipids, including S1P and prostacyclin analogs, and has been shown to protect the endothelium while suppressing platelet aggregation and inflammatory responses both in vitro and in vivo.^45^ Further research is needed to delineate the specific functions of the respective components; however, this approach holds great promise for therapeutic applications in conditions that are characterized by inflammation and thrombosis^45^ and perhaps also in patients with CKD.

We acknowledge several limitations of this study. The inclusion of a healthy control group would have allowed for more accurate comparisons, particularly in milder stages of CKD.

In addition, we were unable to establish definitive causal relationships due to the observational study design.

A notable strength of our study is the validation of our findings in an independent cohort, the comprehensive clinical characterization of patients at baseline allowed us to effectively adjust for potential confounders.

In conclusion, the present study provides novel evidence that circulating APOM levels are independently associated with an increased risk of major atherosclerotic cardiovascular events in nondialysis patients with CKD. Although the precise underlying mechanism remains to be fully clarified, the anti-inflammatory and vascular-protective properties of APOM/S1P are likely contributors to this association. These findings may offer a promising direction for future research aimed at developing targeted therapeutic strategies to address cardiovascular complications in patients with CKD.

## ARTICLE INFORMATION

### Sources of Funding

This research was funded by the Austrian Science Fund (FWF) apoA-I Mimetic Peptide Lipid Assemblies (grant doi: 10.55776/I5703), and the Augustinus Foundation, Denmark. The CARE FOR HOMe study (Cardiovascular and Renal Outcome in CKD 2–4 Patients–The Fourth Homburg Evaluation) was supported by a grant from Else Kröner-Fresenius-Stiftung. For open access purposes, the author has applied a CC-BY public copyright license to any author-accepted manuscript version arising from this submission.

### Disclosures

None.

### Supplemental Material

Tables S1–S5

Figures S1–S2

Major Resources Table
